# Perceived Waist-to-Hip Ratio Predicts Attractiveness, Age, and Parity Judgments in Pre-Contemporary European Portraits of Clothed Women

**DOI:** 10.1007/s10508-026-03485-3

**Published:** 2026-06-30

**Authors:** Jeanne Bovet, Valentine Truchard, Charlotte Touzeau, Coralie Chevallier, Nicolas Baumard

**Affiliations:** 1 School of Psychology, Northumbria University, Newcastle Upon Tyne, NE1 8ST UK; 2 Institut Jean Nicod, Département d’études Cognitives, Ecole Normale Supérieure, Université PSL, EHESS, CNRS, Paris, France

**Keywords:** Attractiveness, Reproductive cues, Historical portraits, Body perception, Parity, Age

## Abstract

Women with a lower waist-to-hip ratio (WHR) are consistently judged as more attractive, a finding replicated across diverse populations and methods. From an evolutionary perspective, this preference may reflect selection for cues of female reproductive potential, as WHR reliably tracks both age and parity. However, virtually all previous studies present nude or minimally clothed bodies in highly standardized conditions, leaving open whether WHR-based judgments generalize to more ecologically valid settings and whether they extend beyond attractiveness to the reproductive traits WHR is a cue of. We addressed both questions by analyzing 608 painted female figures from European portraits (1650–1950), depicting fully clothed women in visually complex and minimally sexualized settings. Online Prolific observers (N = 1,525) independently estimated the body shape, attractiveness, age, and likelihood of previous childbirth from either the body or the face of the painted characters. Perceived WHR strongly predicted all three trait judgments: lower WHR was associated with higher attractiveness, younger perceived age, and lower perceived likelihood of previous childbirth. These relationships persisted after controlling for posture, body orientation, perceived weight, perceived facial age, artistic school, and date of the artwork. Our findings show that WHR-based perceptual heuristics are not artefacts of the narrow stimulus conditions used in prior research but persist in visually complex, clothed, and historically diverse depictions of women. They further suggest that fashion styles that exaggerate WHR may have functioned as strategic amplifiers of reproductively relevant cues, with implications for theories of mate choice, social signaling, fashion history, and cultural evolution.

## Introduction

The ratio between the waist and the hips circumferences (Waist-to-Hip Ratio, or WHR) has been one of the most extensively studied bodily cues in research on physical attractiveness. Women with a low WHR are consistently judged more attractive than those with higher WHRs. This finding is one of the most widely replicated results in research on physical attractiveness. It has been documented across a broad range of populations and methodologies, including samples from China, England, Germany, Papua New Guinea, Poland, and New Zealand, the United States (e.g., Dixson et al., 2007; Kościński, 2014; Pazhoohi et al., 2020; Platek & Singh, 2010; Singh, 1993a; Sorokowski et al., 2014; Weeden & Sabini, 2005; White et al., 2020). From an evolutionary perspective, the link between WHR and attractiveness has been proposed to reflect an adaptive preference: because WHR is a reliable indicator of female age and parity (two key components of reproductive potential), observers who attended to WHR would have gained probabilistic information about a potential mate’s reproductive value.

### Waist-to-Hip Ratio as a Cue to Reproductive Potential

The adaptive interpretation of the WHR preference rests on the claim that WHR carries reliable information about female mate value. Several early hypotheses, such as WHR as a cue of health or a cue of maternal behaviour, have received limited theoretical or empirical support (reviewed in Bovet, 2019). By contrast, there is robust evidence that WHR is a reliable indicator of two variables directly tied to a woman’s residual reproductive potential: age and parity. Fertility is correlated with age in women (Dunson et al., 2002; Fretts et al., 1995; Menken & Larsen, 1986; Wesselink et al., 2017), and each previous birth reduces both the number of potential future children and the investment available for them, even when age is controlled for (Kiely et al., 1986; Merklinger‐Gruchala et al. 2015). Furthermore, WHR is the only reliable external cue of current pregnancy (a state associated with null current fertility), which may have added further selective pressure favouring attention to this bodily dimension (Bovet, 2019).

If this adaptive account is correct, the association between WHR and attractiveness should be general and robust, not an artifact of particular experimental conditions. Moreover, observers might use WHR to infer not just attractiveness but also the underlying traits it is a cue of, including age and parity. We address both of these predictions below.

### How Robust Is the WHR–Attractiveness Link?

Despite the extensiveness of the evidence supporting a preference for low WHR, two lines of criticism have questioned the generalizability of this link. The first concerns the role of potential confounds, most notably body mass index (BMI). Because WHR and BMI are correlated in natural bodies, and because many experimental stimuli manipulate waist size in ways that simultaneously alter apparent body mass, several authors have argued that BMI or related measures may be stronger predictors of attractiveness than WHR (Brooks et al., 2010; Furnham et al., 2005; Tassinary & Hansen, 1998; Tovée et al., 1999). The second concerns cross-cultural variability. While the great majority of studies report preferences for lower WHRs, research among several subsistence farming, horticultural and hunter-gatherer populations has sometimes reported preferences for higher WHRs (Dixson et al., 2007; Sugiyama, 2004; Wetsman & Marlowe, 1999; Yu and Shepard Jr. 1998). Notably, some of these discrepancies appear to depend on stimulus design. For example, initial work among Hadza hunter–gatherers using front-posed stimuli suggested a preference for higher WHR (Wetsman & Marlowe, 1999), whereas a later replication using profile-view stimuli found a preference for lower WHR values (Marlowe et al., 2005). Although adaptive variation in mate preferences across ecologies has been proposed (Marcinkowska et al., 2019), a pattern that is highly sensitive to methodological details would be difficult to reconcile with the notion of a broadly evolved perceptual bias.

These debates highlight that the theoretical significance of the WHR preference depends on its generalizability. And this, in turn, raises a critical question: could the existing evidence rest on methodological features shared by virtually all prior studies?

### A Shared Methodological Blind Spot

A review of the WHR–attractiveness literature reveals that previous studies, despite their diversity in other respects, share three characteristics that may systematically inflate the apparent role of WHR.

First, stimuli almost universally represent women with no or minimal clothing (see Table 1 for a summary). Stimuli include outlines, drawings, avatars, photographs or body scans of naked women, or of women in underwear, bikini, swimsuit, or tight leotards. A small number of studies included a few clothed figures alongside nude stimuli (Henss, 2000; Suschinsky et al., 2007; Zotto & Pegna, 2017), but none analysed attractiveness ratings for clothed stimuli separately. While nudity or minimal clothing maximises the visibility of body shape, it also introduces an important confound: stimuli that present the female body in overtly sexualized contexts are known to elicit heightened sexual attention and to modify visual, cognitive and neural processing in ways that may amplify the salience of body-shape cues (Bernard et al., 2012; Cundall & Guo, 2017; Gervais et al., 2012, 2013; Hietanen & Nummenmaa, 2011; Zotto & Pegna, 2017). It therefore remains unknown whether WHR is also used in more ecologically typical scenarios where women are fully clothed and not directly sexualized.

**Table 1:** Examples of stimulus types used in the WHR–attractiveness literature

Image type	Clothing	Examples of references
Drawings		
	Nude	Dixson, et al. (2007a, 2007b, 2010a, 2010b, 2010c); Swami et al. (2009)
	Leotard	Furnham et al. (2006); Hübner and Ufken (2024); Schützwohl (2006); Singh (1993a, 1993b, 1994); Tassinary and Hansen (1998); Tovée et al. (1999, 2002)
Photographs		
	Nude	Perilloux et al. (2010)
	Underwear/Bikini	Andrews et al. (2017)
	Leotards	Rilling et al. (2009); Swami et al. (2006); Swami and Tovée (2005, 2006)
Manipulated photographs		
	Nude	Dixson et al. (2010a, 2010b, 2010c, 2011); Garza et al. (2016)
	Underwear/Bikini	White et al. (2020); Wilson et al. (2005)
	Leotards	Puhl and Boland (2001); Rozmus-Wrzesinska and Pawlowski (2005)
	Mixed	Furnham and Reeves (2006); Henss (2000); Suschinsky et al. (2007)
Photographs before/after cosmetic surgery		
	Nude	Dixson et al. (2010a, 2010b, 2010c, ); Platek and Singh (2010); Singh and Randall (2007)
Computer-generated images		
	Nude	Bovet et al. (2016)
	Underwear/Bikini	Brooks et al. (2010); Crossley et al. (2012); Kościński (2014); Sorokowski et al. (2014)
	Mixed	Pazhoohi et al. (2020); Zotto and Pegna (2017)

WHR, waist-to-hip ratio. The list of references is non-exhaustive

Second, most studies use a small number of stimuli, typically between 6 and 18 (e.g., Dixson et al., 2010a, 2010b, 2010c; Henss, 2000; Singh, 1993a, 1993b; Singh & Randall, 2007; Swami et al., 2009; Tassinary & Hansen, 1998), although a few studies use larger sets (between 50 and 200, e.g., Donohoe et al., 2009; Kościński, 2014; Tovée et al., 1999; Tovée & Cornelissen, 2001). Even when using larger numbers of stimuli, or allowing the participants to change several dimensions of an avatar, effectively generating an even greater stimulus set (Brooks et al., 2010; Crossley et al., 2012), those studies often use one or a small number of base images or model “identities” which is then digitally transformed, limiting the generalisation of the results to different models. Moreover, the same stimuli are often reused across multiple studies, resulting in a form of pseudoreplication (Bovet et al., 2022).

Third, stimuli are all highly standardized: identical outfit, posture, and laboratory or studio conditions. While standardisation is designed to isolate the variable of interest, it also removes the visual complexity of real-world settings and directs the observers’ attention toward the WHR during the study, potentially inflating effect sizes relative to what would be detectable in more ecologically valid contexts.

Taken together, these three features mean that the entire evidence base for the WHR–attractiveness link comes from stimuli that are nude or near-nude, small in number, and artificially homogeneous. If WHR-based attractiveness judgments depend on any of these conditions, then the strength and generality of the association (and by extension its evolutionary interpretation) may have been overstated.

### The Present Study

In the course of a separate project investigating historical changes in body shape depictions across European art (https://osf.io/v53ag), we compiled a large database of painted figures from French and English portraits spanning three centuries (1650–1950). These two countries offer the most abundant and continuous corpus of painted portraits, particularly full-length representations, prior to the contemporary era. We realised that this dataset also offered a unique opportunity to test the generalisability of WHR-based judgments, because the stimuli differ from those used in prior WHR–attractiveness research on every one of the dimensions identified above: they depict fully clothed women, in large numbers (N > 600), drawn from distinct identities, and embedded in socially rich, visually complex, and minimally sexualized settings.

Our first goal is to test whether the well-established WHR–attractiveness link holds in this markedly different context. If the association persists under conditions that remove every methodological feature shared by previous studies, it would provide strong evidence that WHR-based attractiveness judgments are not merely an artifact of narrow stimulus conditions, and would substantially strengthen the case for WHR as a general perceptual cue.

Our second goal is to test whether WHR also guides perceptions of age and parity. If the adaptive account is correct (that is, if WHR preferences exist because WHR carries information about reproductive potential), then observers might not only find lower WHR more attractive but also infer age and parity from it. And while WHR is reliably correlated to reproductive age and parity (Beall & Goldstein, 1992; Bohler et al., 2010; Butovskaya et al., 2017; Lassek & Gaulin, 2006; Seidell et al., 1990; Wells et al., 2011), making it a reliable cue, little research has directly examined whether observers infer these traits from WHR, and none has done so in ecologically valid, clothed depictions. Demonstrating such links would strengthen the interpretation of WHR as a general reproductive heuristic, rather than a purely aesthetic cue.

While a small number of studies have examined WHR in artworks, their research questions and approach differ substantially from ours. For example, Swami et al. (2007a, 2007b) measured women’s WHR in 23 paintings by a single painter (Rubens) and Swami et al. (2007a, 2007b) used manipulated versions of painted nude women as stimuli. Some of our own work has investigated historical changes in WHR depictions using the same dataset presented here (https://osf.io/v53ag), or explored other types of artworks (Bovet & Raymond, 2015). However, no previous research has tested whether WHR predicts attractiveness, age, and parity judgments in a large, fully clothed, and diverse sample of women.

We predict that perceived WHR will be negatively associated with attractiveness ratings, and positively associated with perceived age and perceived likelihood of having ever given birth.

## Method

All hypotheses, target sample sizes, and analytic plans were preregistered on the Open Science Framework (OSF; https://osf.io/v53ag) before data collection. The preregistered protocol was made in two steps and includes both male and female painted characters, as well as some historical analyses which are presented elsewhere (manuscript under review, preregistration and data available on the OSF; https://osf.io/v53ag); here, we report only the female subset. Complete versions of all surveys, data and code are archived on the OSF.

### Painted Characters

Paintings were retrieved through systematic searches of three online public catalogues: The Web Gallery of Art (WGA), the National Portrait Gallery (NPG), and The Metropolitan Museum of Art (Met). These databases were selected because they provide extensive catalogues of European portraiture with standardized metadata and high-resolution digital reproductions. Eligible paintings were required to meet pre-specified criteria. First, works had to be produced between 1650 and 1950. Second, paintings had to depict at least one adult, upper‑class woman with her torso (chest, waist, hips) at least partly visible, such that body shape could plausibly be inferred from clothing contours. From each eligible painting, individual characters were included if they were of a resolution sufficient to distinguish the clothing from the environment. Characters were excluded if they were wearing attire adhering to non-fashion codes (e.g., clerical vestments, legal robes, folk costume, masquerade dress), as these garments could systematically obscure or distort body shape in ways unrelated to fashion norms. Within each database, all characters meeting these criteria were included; selection was therefore based on explicit inclusion rules rather than subjective aesthetic judgment or random sampling from a larger eligible pool.

The final dataset contained 608 female characters drawn from 486 paintings. Of these painted characters, 463 were from French‑school artists and 145 from English‑school artists. A complete stimuli catalogue, including artist, title, date, source and current location, is available on the OSF.

**Fig. 1:**
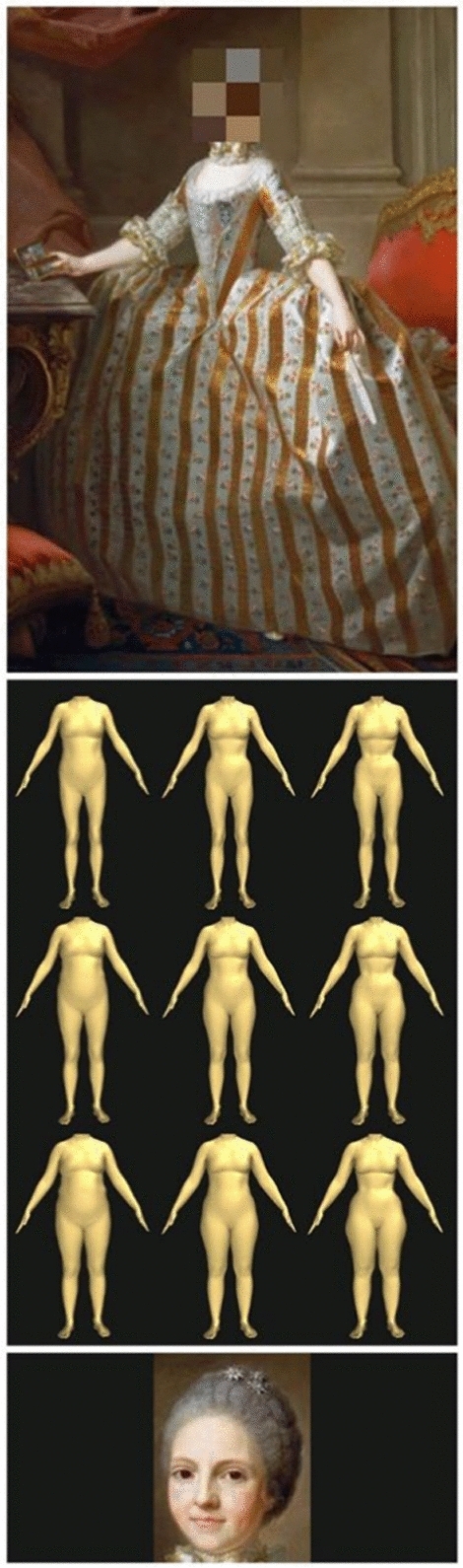
Example of stimuli for the body condition (top) and the face condition (bottom). Portrait of Maria Luisa of Parma by Laurent Pécheux (1765). In the body-shape estimation task, participants were asked to pick the reference silhouette (middle) that best corresponded to the character’s body. The silhouettes crossed three body‑mass levels (50, 65, and 80 kg from top to bottom) with three WHR levels (1.00, 0.70, and 0.40 from left to right)

Each painting was cropped to isolate a single focal female character. For body-based tasks, the face was pixelated to remove facial cues and ensure that judgments were based on body information. For each character with a visible face (N = 542), an additional face-only crop was created for use in the face-based task (see Fig. 1).

### Participants and Procedure

Raters (N = 1,570) were recruited online via Prolific (www.prolific.co), were ≥ 18 years old, living in the UK, fluent in English, and compensated £9 per hour. Participants were randomly assigned to one of three between-subjects tasks: (1) Body‑shape estimation task, (2) Body-based trait ratings, or (3) Face-based trait ratings. After providing informed consent, participants received instructions specific to their assigned task. Each participant viewed a random subset of female characters (between 20 and 55 stimuli, depending on the task) with a randomized presentation order. The study employed a between-subjects design, such that each participant completed only one of the three tasks. This design choice was made to prevent carryover effects in either direction. Exposure to the explicit WHR comparison silhouettes used in the body-shape estimation task could have sensitised participants to waist–hip variation and thereby influenced attractiveness, age, or parity judgments. Conversely, priming participants with questions about age or parity before the estimation task could have biased their silhouette selections independently of perceived WHR. Using independent participant groups for each task ensures that neither form of contamination can occur. Stimuli and response interfaces were delivered online via Qualtrics and data collected in March 2024 and June 2025.

### Measures

#### Body‑Shape Estimation

Participants in the body-shape estimation condition were shown each painted character alongside nine reference silhouettes generated with the Body Shape Visualizer (Perceiving Systems Department, Max Planck Institute for Intelligent Systems). The silhouettes crossed three body‑mass levels (50, 65, 80 kg) with three WHR levels (0.40, 0.70, 1.00); height and other anthropometrics were held constant (see Fig. 1 and the OSF for full measurements). The raters were instructed to click on the body which “best corresponds to this woman”.

For each painted character, the mean WHR and mean body mass of selected silhouettes were calculated across raters. These aggregated values were used as estimates of perceived WHR and perceived body weight. This method, adapted from Bovet and Raymond (2015), provides a continuous estimate of perceived WHR and body weight from 2D, non-standardized images where women’s posture and orientation vary, preventing direct landmark-based measurements.

#### Body-Based Trait Ratings

In the body-based trait rating condition, participants evaluated each character’s attractiveness on a seven-point Likert scale, indicated whether they believed the woman had “never had any children” or “had given birth to at least one child”, and estimated her age on a slider ranging from 15 to 70 years. Because faces were obscured, these judgments were based solely on body information.

#### Face-Based Trait Ratings

Participants in the face-based condition evaluated cropped face images without body information (see Fig. 1). They rated attractiveness (7-point scale) and estimated age (15–70 slider). These ratings were used as covariates in models examining body-based effects.

### Statistical Analyses

All analyses were conducted in R (version 4.5.0). Preregistered mixed-effects models included random intercepts for both stimulus ID and rater ID. Separate models were run for attractiveness, perceived age, and perceived likelihood of previous childbirth. Robustness checks included pre-registered and additional covariates for perceived body weight, posture, orientation, artistic school, database of origin, facial age, facial attractiveness, and artwork date.

## Results

### Descriptive Analyses

After screening for completion time and missing data, 36 incomplete responses were removed (less than 3% of participants who clicked on the survey link), and nine additional respondents (less than 1%) were excluded for implausibly short completion times (under 4 min, compared to an overall median completion time of 9 min). The final sample included 1,525 respondents (760 women, 750 men, 12 non-binary, 3 undisclosed; see Table 2 for details).

**Table 2:** Demographic information of raters by task

		Gender					
Task	*N*	Men	Women	Non-binary	Undisclosed	Age *M* (*SD*)	Age range
Body-shape estimation	593	293	297	2	1	46 (14)	18–81
Body-based trait ratings	661	322	327	10	2	45 (14)	18–80
Face-based trait ratings	271	135	136	0	0	43 (14)	21–81

On average, each painted character’s body shape was estimated by 51 raters, whereas the body-based and the face-based trait judgments were each provided by an average of 20 raters per character. Cumulative-mean trajectories indicated that the mean estimates stabilized (± 5% of the full scale) after roughly 25 raters for body-shape measurements and after about 20 raters for trait ratings. Additional respondents contributed only negligible precision gains beyond those thresholds (results available on the OSF).

The full sample included 608 painted female characters. The mean perceived WHR was 0.67 (SD = 0.08), the mean perceived age from bodies was 35.8 years (SD = 7.08), the mean body attractiveness rating was 4.09 (SD = 0.75) on a 1–7 scale and the likelihood of being perceived as having given birth before was 0.55 (SD = 0.24). For the subset with facial data (n = 542), mean perceived facial age was 35.6 years (SD = 9.16), and mean facial attractiveness rating was 3.55 (SD = 0.81). Table 3 presents descriptive statistics.

**Table 3:** Descriptive statistics for mean perceived traits aggregated at the character level

Variable	*N*	*M*	*SD*	Median	Range
Waist-to-hip ratio	608	0.67	0.08	0.68	0.47–0.92
Body attractiveness	608	4.09	0.75	4.11	2.11–6.19
Body age	608	35.80	7.08	34.43	22.40–61.06
Likelihood of previous childbirth	608	0.55	0.24	0.58	0–1
Facial attractiveness	542	3.55	0.81	3.52	1.42–5.80
Facial age	542	35.59	9.16	33.55	17–68.57

Values are means (*M*), standard deviations (*SD*), medians, and observed ranges aggregated at the character level

We first examined correlations between character-level mean ratings of perceived attractiveness, age, and parity. All pre-registered correlations were computed at the stimulus level, using mean ratings aggregated across raters for each painted character. Perceived body attractiveness was strongly negatively correlated with perceived body age (*r* =  − .79, *p* < .001) and moderately negatively correlated with perceived body parity (*r* =  − .53, *p* < .001), indicating that women rated as more attractive were also perceived as younger and less likely to have previously given birth. Perceived age and perceived parity were strongly positively correlated (*r* = .69, *p* < .001), suggesting that older-looking women were more often judged as parous.

We then examined whether face-based and body-based ratings of the same dimension reflected similar or distinct perceptual information, again at the stimulus level. For attractiveness, body and face ratings were positively but only moderately correlated (*r* = .33, *p* < .001), and the same was true for age (*r* = .48, *p* < .001). These modest correlations suggest that while face and body cues carry partially overlapping information, they nonetheless constitute meaningfully distinct sources of perceptual information about a woman’s attractiveness and age.

**Fig. 2:**
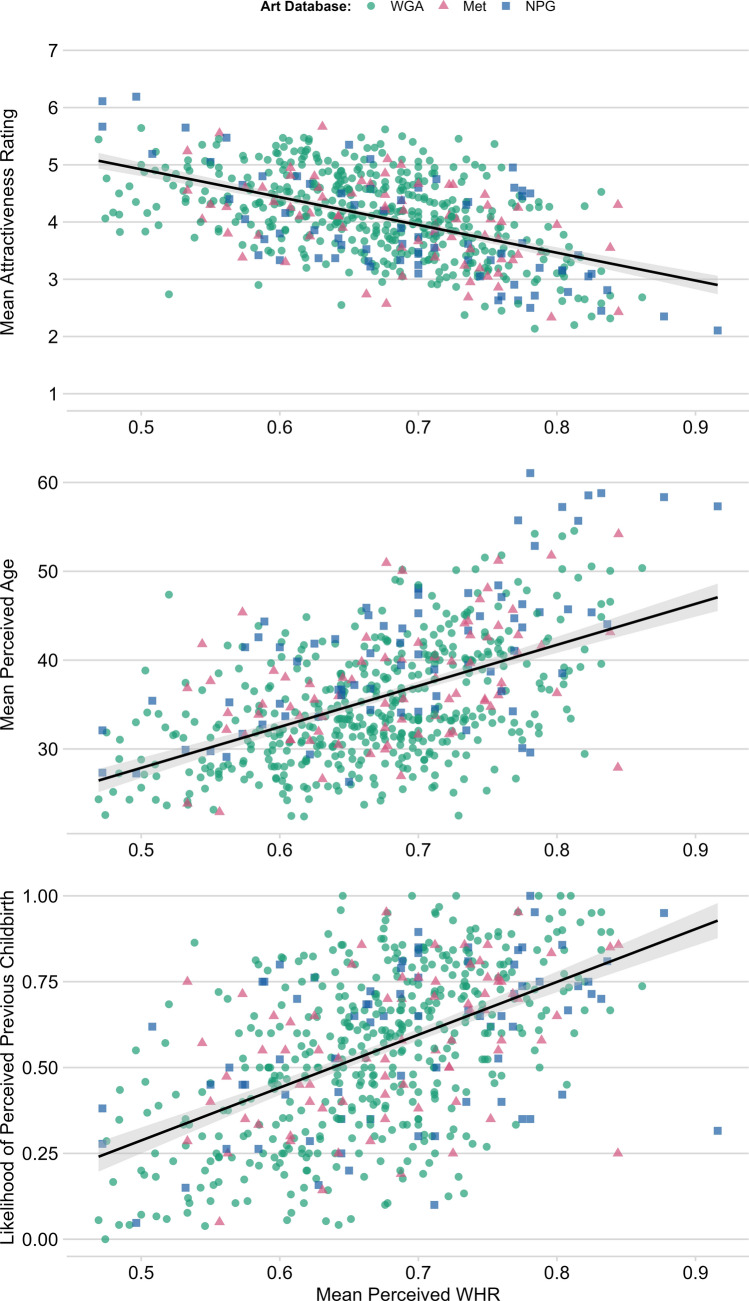
Relationships between perceived WHR and attractiveness (top), perceived age (middle) and likelihood of perceived previous childbirth (bottom) for women depicted in portraits. Dots show the mean WHR and mean estimated trait for each character (dot shapes distinguish databases: WGA = Web Gallery of Art; Met = Metropolitan Museum of Art; NPG = National Portrait Gallery). Lines depict ordinary‑least‑squares fits; shaded ribbons indicate the ± 95% confidence interval. Women with lower WHRs are perceived as more attractive, younger, and less likely to have ever given birth than women with higher WHRs

Three separate pre-registered mixed-effects models, including random intercepts for both rater and stimulus IDs, were run to analyse the associations between WHR (estimated as the mean WHR of all silhouettes selected for each character), and ratings of attractiveness, age and parity (from a participant set separate from body shape estimations). Figure 2 visualises these relationships, aggregated at the character level.

### Attractiveness

For body attractiveness (1–7 scale), the linear mixed-effects model indicated that WHR was a strong negative predictor: *b* = − 4.82, SE = 0.30, *t*(703) = − 15.83, *p* < 0.001. Expressed per 0.10-unit increase in WHR (a scale more interpretable over the observed range), attractiveness declined by 0.48 scale points, 95% CI [− 0.54, − 0.42].

A reviewer noted that the literature sometimes suggests an optimal WHR of around 0.7 for female attractiveness (Singh, 1993b), raising the question of whether the relationship between WHR and attractiveness might be curvilinear rather than strictly linear. To address this, we fitted an exploratory mixed-effects model including both a linear and a quadratic WHR term (with random intercepts for stimulus and rater). Both the linear term (*b* = 9.74, SE = 3.74, *t*(768) = 2.60, *p* = .009) and the quadratic term were significant (*b* =  − 10.96, SE = 2.81, *t*(765) = −3.91, *p* < .001), indicating an inverted-U relationship. However, it should be noted that the estimated peak WHR (0.44) falls below the minimum observed WHR in our sample (0.47), meaning that the ascending portion of the inverted-U is not directly observed in the data. The curvilinear model therefore largely captures the same negative trend as the linear model, and the estimated optimum should be interpreted with caution as it relies on extrapolation beyond the observed range.

### Perceived Age

For perceived age, the corresponding model returned a positive WHR effect: *b* = 42.54, SE = 2.84, *t*(730) = 14.97, *p* < 0.001. Each increment in 0.10 WHR increased estimated age by 4.25 years, 95% CI [3.70, 4.81].

### Perceived Parity

Parity judgments (0 = no previous childbirth; 1 = at least one previous childbirth) were analysed with a binomial logistic mixed model. WHR positively predicted the log-odds of being classified as parous: β = 7.75, SE = 0.51, *z* = 15.12, *p* < 0.001. A 0.10-unit increase in WHR increased the odds by a factor of 2.17, 95% CI [1.96, 2.40]. As a robustness check, we re-estimated the parity model after excluding paintings that depicted children (N = 23), and the results remained virtually identical (see Model 9 in Table 4).

**Table 4:** Results of multilevel models predicting attractiveness, perceived age, and perceived likelihood of previous childbirth

	Attractiveness			Perceived age			Perceived likelihood of previous childbirth		
	Model 1	Model 2	Model 3	Model 4	Model 5	Model 6	Model 7	Model 8	Model 9
(Intercept)	7.332*** (0.208)	8.939*** (0.994)	8.809*** (0.977)	7.197*** (1.931)	− 31.170*** (8.654)	− 29.240*** (8.244)	− 4.945*** (0.347)	− 6.117*** (1.466)	− 7.793*** (0.456)
WHR	− 4.820*** (0.304)	− 3.232*** (0.388)	− 3.301*** (0.382)	42.541*** (2.843)	23.702*** (3.383)	19.941*** (3.279)	7.747*** (0.512)	3.140*** (0.618)	3.110*** (0.597)
Database (NPG)		− 0.022 (0.128)	− 0.085 (0.127)		3.168** (1.151)	1.833 + (1.105)		− 0.233 (0.197)	− 0.242 (0.184)
Database (WGA)		0.198* (0.089)	0.149 + (0.088)		− 0.722 (0.800)	− 1.473 + (0.765)		− 0.242 + (0.137)	− 0.220 + (0.123)
School (French)		− 0.113 (0.079)	− 0.080 (0.078)		0.108 (0.715)	0.381 (0.679)		− 0.017 (0.121)	− 0.070 (0.115)
Sitting		− 0.043 (0.052)	− 0.039 (0.051)		0.748 (0.473)	0.553 (0.449)		0.034 (0.080)	0.001 (0.077)
Front View		0.030 (0.056)	0.024 (0.055)		0.229 (0.507)	0.143 (0.481)		0.022 (0.086)	0.027 (0.082)
Facial Age		− 0.024*** (0.003)	− 0.015*** (0.003)			0.236*** (0.030)		0.026*** (0.005)	0.017*** (0.004)
Body Weight		− 0.019*** (0.005)	− 0.017** (0.005)		0.303*** (0.048)	0.319*** (0.046)		0.093*** (0.009)	0.091*** (0.008)
Date of Artwork		− 0.000 (0.001)	− 0.001 + (0.001)		0.021*** (0.005)	0.014** (0.004)		− 0.001 + (0.001)	
Facial Attractiveness			0.181*** (0.038)		− 1.981*** (0.291)	− 0.563 + (0.330)		0.080 (0.059)	
*Random effects*									
*SD* (Intercept Rater)	0.735	0.743	0.743	4.104	4.016	4.016	0.524	0.519	0.531
*SD* (Intercept Stimuli)	0.579	0.544	0.531	5.620	5.007	4.713	0.871	0.751	0.685
*SD* (Observations)	1.020	1.017	1.017	8.204	8.113	8.113			
*N* (Observations)	12,865	11,507	11,507	12,865	11,507	11,507	12,865	11,507	11,041

Coefficients are unstandardized (*B*) with standard errors in parentheses. WHR, waist-to-hip ratio; NPG, National Portrait Gallery; WGA, Web Gallery of Art

+ *p* < .10. * *p* < .05. ** *p* < .01. *** *p* < .001

### Robustness Checks

Robustness checks confirmed that the WHR effect was not simply an artifact of other stylistic or contextual covariates. Each model was re-estimated with both preregistered and additional controls: database of origin (WGA, NPG, Met), artistic school (French vs. English), posture of the character (sitting/bending vs. standing), body orientation (front-view vs. other), mean facial age and attractiveness (based on independent estimations from the face only), perceived body weight and date of the artwork (allowing us to account for historical variation in fashion and painting style). These models (reported in Table 4) show that WHR effects on all perceived traits remained significant (all *p* < 0.001).

### Observers’ Gender

To examine whether WHR-based judgments differed by rater gender, we estimated exploratory mixed-effects models including a WHR × gender interaction term (with random intercepts for stimulus and rater), restricting the sample to observers identifying as women or men, as the remaining gender categories had insufficient numbers of raters for reliable estimation. For attractiveness ratings, the interaction was significant (*b* = 0.86, *SE* = 0.24, *t*(11690) = 3.58, *p* < .001). Among men raters, higher WHR strongly predicted lower attractiveness (*b* =  − 5.27, *SE* = 0.33, *t*(935) = −15.97, *p* < .001). Among women, the association remained negative but was somewhat attenuated (*b* =  − 5.27 + 0.86 =  − 4.41), indicating that although both genders perceived higher WHR as less attractive, men were slightly more sensitive to WHR variation when evaluating attractiveness. The main effect of gender was significant (*b* = 0.40, *SE* = 0.06, *t*(647) = 6.76, *p* < .001), indicating that women raters gave overall higher attractiveness ratings than men. For perceived age, neither the main effect of gender nor the WHR × gender interaction was significant (all *p* > .07), indicating that men and women did not differ either in their baseline age estimates or in their reliance on WHR as an age cue. Similarly, for parity judgments, neither the main effect of gender nor the interaction term reached significance (all *p* > .2). Thus, gender differences emerged only for attractiveness ratings, both in overall evaluative tendencies and in the magnitude of the WHR effect. Full model results are reported on the OSF.

In sum, and in line with our predictions, a lower (i.e., more “curvy”) WHR in painted characters was associated with higher attractiveness ratings, younger perceived age, and a lower perceived likelihood of previous childbirth.

## Discussion

We tested two predictions. First, whether the well-established association between low WHR and high attractiveness generalizes beyond the narrow stimulus conditions shared by virtually all prior studies. Second, whether observers also use WHR to infer age and parity, the reproductive traits that WHR actually tracks. Our results support both predictions. In a large and diverse sample of fully clothed women from European portraits (1650–1950), a lower perceived WHR was significantly associated with higher attractiveness ratings, younger perceived age, and a smaller likelihood of being perceived as having ever given birth. These effects remained significant even when controlling for potential confounds, such as perceived weight, posture, body orientation, artistic school, and date of the artwork.

The persistence of the WHR–attractiveness link in our stimuli is notable, given how different they are from those used in prior research. A robust body of evidence has shown that lower WHR is consistently judged as more attractive across cultures and methods (e.g., Dixson et al., 2007; Kościński, 2014; Pazhoohi et al., 2020; Platek & Singh, 2010; Singh, 1993a; Sorokowski et al., 2014; Weeden & Sabini, 2005; White et al., 2020). However, this evidence comes almost entirely from nude or minimally clothed, highly standardized stimuli presented in sexualized contexts; conditions likely to amplify the salience of body-shape cues (Bernard et al., 2012; Gervais et al., 2012, 2013; Hietanen & Nummenmaa, 2011; Zotto & Pegna, 2017). Our stimuli remove all of these features: the women are fully clothed in historical costumes, vary in posture and orientation, and are embedded in socially rich, minimally sexualized settings. The fact that WHR effects persist under these conditions indicates that they are not artefacts of the narrow stimulus conditions used in prior research and that WHR remains a psychologically salient cue in visually complex, ecologically valid contexts. As such, our findings complement recent speed-dating evidence that WHR predicts women’s attractiveness ratings in live, face-to-face interactions (Sidari et al., 2021).

The associations between WHR and perceived age and parity are, in some respects, the more novel contribution of this study. Although a preference for low WHR has long been proposed to reflect selection for cues of reproductive potential (reviewed in Bovet, 2019), surprisingly little research has tested whether observers actually infer reproductive traits from WHR. Evidence concerning WHR and youthfulness has been mixed, partly because WHR follows a non-linear trajectory across the lifespan: relatively high in childhood, decreasing at puberty, and rising again after peak fertility years (Bovet, 2019). Depending on where the depicted woman falls along this U-shaped curve, observers may associate lower WHR with either youth or maturity, which could explain divergent findings across previous studies. Our data show a clear positive association between perceived WHR and perceived age across a wide range of painted women. As for parity, only one prior study had documented an association between higher WHR and higher perceived parity (Andrews et al., 2017). Our results, therefore, provide the strongest evidence to date that WHR is associated with perceived reproductive stage and residual reproductive potential in diverse and complex depictions of women (note: the perceived age of the characters ranges from 17 to 69 years old). This supports the interpretation of WHR as a general reproductive heuristic, not merely an aesthetic preference.

Our results also inform the long-standing debate about the relative importance of WHR compared to other body metrics such as BMI. Several authors have argued that WHR may be secondary to preferences for body adiposity or other shape parameters (Brooks et al., 2010; Furnham et al., 2005; Tassinary & Hansen, 1998; Tovée et al., 1999). In our dataset, WHR was not experimentally manipulated and was not made salient by stimulus design; it was one of many visual cues available to observers. Yet perceived WHR robustly predicted attractiveness, perceived age, and perceived parity even after controlling for perceived body weight. This suggests that WHR has an independent perceptual role in naturalistic contexts and is not merely a by-product of adiposity cues.

These results have broader implications for theories of mate choice, cultural evolution, and signaling. From a mate choice perspective, the persistence of WHR effects in fully clothed depictions suggests that the WHR might be used as a generalized cue to attractiveness and reproductive potential, used in diverse social contexts, not solely in sexual scenarios. In cultural evolutionary terms, our results could explain the widespread adoption and enduring popularity of physically restrictive attire such as corsets, farthingales, panniers or crinolines. By exaggerating waist–hip contrast, these garments may have exploited perceptual biases linked to WHR. Accordingly, while our stimuli are naturalistic in that they reflect real historical appearances, the WHRs perceived by observers reflect not only underlying morphology but also culturally constructed silhouettes designed to enhance specific visual cues. Importantly, such garments were systematically associated with particular historical periods (Steele, 2001; Vigarello, 2004; Waugh, 2015). However, the associations we report cannot be reduced to period-specific fashion effects, as WHR remained a strong predictor of attractiveness, perceived age, and perceived parity even when controlling for the year of production of the artwork (i.e., historical period). While a costly signal hypothesis explains elaborate clothing as a display of wealth (Veblen, 1965), it does not fully account for the specific shapes these garments imposed. From a signaling perspective, such silhouettes may have functioned as an extended phenotype, artificially enhancing traits linked to reproductive potential (Davis & Arnocky, 2022). Like cosmetics, photo filters, or even gendered products (Borau & Bonnefon, 2020; Etcoff et al., 2011), historical garments may have strategically amplified preferred traits.

A first limitation concerns the cultural and demographic scope of our study. Paintings were European portraits depicting White women. As a result, the generalisability of our findings to other cultural, ethnic, and geographic contexts remains uncertain. It is possible that both the perception of WHR and its association with attractiveness and perceived reproductive traits vary as a function of observer–target ethnic matching or cultural familiarity (Ridley et al., 2025). Because we did not record participants’ racial or ethnic background, we were unable to test whether such factors moderated the associations observed here. Similarly, the corpus reflects the aesthetic preferences of social elites and thus provides little information about body representations among other social strata. Future research should therefore examine different stimuli sets, including non-European and non-White populations, and explicitly test the role of participant demographics in shaping WHR-based judgments.

Another limitation of the present study is that we did not collect information about raters’ sexual orientation or relationship status. From an evolutionary perspective, inferences about a woman’s reproductive potential may be especially relevant for single heterosexual men, for whom such cues could directly inform mate selection decisions. Our exploratory analyses of rater gender indicate that the association between WHR and attractiveness was somewhat stronger among male raters, consistent with this idea. At the same time, there are theoretical reasons to expect substantial convergence across groups: WHR may inform not only mate choice but also broader social processes, including intrasexual competition among heterosexual women and general social evaluation (e.g., age inferences are relevant beyond mating contexts). Moreover, shared developmental or learning mechanisms may produce considerable agreement in judgments across genders and sexual orientations. Although empirical evidence directly addressing moderation by sexual orientation and relationship status remains limited, existing findings suggest that sexual orientation may influence the strength of attractiveness judgments, whereas relationship status appears to exert little or no effect (Mitrovic et al., 2016; Rupp et al., 2009; Shiramizu et al., 2020). Because our sample likely included individuals of varying orientations and relationship statuses, any subgroup-specific amplification of WHR effects would have been diluted in the aggregate analyses, meaning our estimates are likely conservative.

Our findings rely on context-dependent perceived traits, which opens several possible explanatory pathways. The association between WHR and perceived age, for example, may reflect multiple, potentially overlapping processes. Observers may directly use WHR as a cue to estimate age, or they may instead draw on other cues, such as clothing style or posture, that also shape body-shape judgments, thereby producing correlated estimates. We adopted several strategies to reduce overlap between cues: Cropping each painting to the focal figure (removing contextual cues), pixelating faces, separating rater groups for body shape versus trait judgments, and including covariates such as posture, perceived body weight, facial age and facial attractiveness in our models. The use of historical portraits may also help minimise reliance on everyday non-body cues, given modern raters’ limited familiarity with historical clothing signals. These experimental and statistical controls show the robustness of the associations we observe and, together with prior research on WHR, support the idea that observers used the WHR when making social judgments. Still, some degree of cue overlap is unavoidable in any naturalistic setting, leaving open the precise causal mechanisms involved.

Future research should directly compare WHR perceptions of the same individuals in clothed versus unclothed conditions, using both historical and contemporary fashions. Such work would allow a clearer quantification of how clothing alters WHR and, in turn, influences judgments of reproductive and social traits. This approach would build on our findings by testing the interplay between evolved preferences, aesthetic norms and fashion-driven body ideals.

## Data Availability

Complete versions of all surveys, data and code are archived on the OSF (https://osf.io/v53ag).

## References

[CR1] Andrews, T. M., Lukaszewski, A. W., Simmons, Z. L., & Bleske-Rechek, A. (2017). Cue-based estimates of reproductive value explain women’s body attractiveness. *Evolution and Human Behavior,**38*, 461–467. 10.1016/j.evolhumbehav.2017.04.002

[CR2] Beall, C. M., & Goldstein, M. C. (1992). High prevalence of excess fat and central fat patterning among Mongolian pastoral nomads. *American Journal of Human Biology,**4*(6), 747–756. 10.1002/ajhb.1310040606 28524633 10.1002/ajhb.1310040606

[CR3] Bernard, P., Gervais, S. J., Allen, J., Campomizzi, S., & Klein, O. (2012). Integrating sexual objectification with object versus person recognition: The sexualized-body-inversion hypothesis. *Psychological Science,**23*(5), 469–471. 10.1177/0956797611434748 22477107 10.1177/0956797611434748

[CR4] Bohler, H., Mokshagundam, S., & Winters, S. J. (2010). Adipose tissue and reproduction in women. *Fertility and Sterility,**94*(3), 795–825. 10.1016/j.fertnstert.2009.03.079 19589523 10.1016/j.fertnstert.2009.03.079

[CR5] Borau, S., & Bonnefon, J.-F. (2020). Gendered products act as the extended phenotype of human sexual dimorphism: They increase physical attractiveness and desirability. *Journal of Business Research,**120*, 498–508. 10.1016/j.jbusres.2019.03.007

[CR6] Bovet, J. (2019). Evolutionary theories and men’s preferences for women’s waist-to-hip ratio: Which hypotheses remain? A systematic review. *Frontiers in Psychology,**10*. 10.3389/fpsyg.2019.01221 10.3389/fpsyg.2019.01221 PMC6563790 31244708

[CR7] Bovet, J., Lao, J., Bartholomée, O., Caldara, R., & Raymond, M. (2016). Mapping female bodily features of attractiveness. *Scientific Reports,**6*, Article 18551. 10.1038/srep18551 26791105 10.1038/srep18551 PMC4726249

[CR8] Bovet, J., & Raymond, M. (2015). Preferred women’s waist-to-hip ratio variation over the last 2,500 years. *PLoS ONE,**10*(4), Article e0123284. 10.1371/journal.pone.0123284 25886537 10.1371/journal.pone.0123284 PMC4401783

[CR9] Bovet, J., Tognetti, A., & Pollet, T. V. (2022). Methodological issues when using face prototypes: A case study on the Faceaurus dataset. *Evolutionary Human Sciences,**4*. 10.1017/ehs.2022.25 10.1017/ehs.2022.25 PMC10426020 37588902

[CR10] Brooks, R., Shelly, J. P., Fan, J., Zhai, L., & Chau, D. K. P. (2010). Much more than a ratio: Multivariate selection on female bodies. *Journal of Evolutionary Biology,**23*(10), 2238–2248. 10.1111/j.1420-9101.2010.02088.x 20840313 10.1111/j.1420-9101.2010.02088.x

[CR11] Butovskaya, M., Sorokowska, A., Karwowski, M., Sabiniewicz, A., Fedenok, J., Dronova, D., et al. (2017). Waist-to-hip ratio, body-mass index, age and number of children in seven traditional societies. *Scientific Reports,**7*(1), Article 1622. 10.1038/s41598-017-01916-9 28487573 10.1038/s41598-017-01916-9 PMC5431669

[CR12] Crossley, K. L., Cornelissen, P. L., & Tovee, M. J. (2012). What is an attractive body? Using an interactive 3D program to create the ideal body for you and your partner. *PLoS ONE,**7*. 10.1371/journal.pone.0050601 10.1371/journal.pone.0050601 PMC3510069 23209791

[CR13] Cundall, A., & Guo, K. (2017). Women gaze behaviour in assessing female bodies: The effects of clothing, body size, own body composition and body satisfaction. *Psychological Research Psychologische Forschung,**81*(1), 1–12. 10.1007/s00426-015-0726-1 26586290 10.1007/s00426-015-0726-1

[CR14] Davis, A. C., & Arnocky, S. (2022). An evolutionary perspective on appearance enhancement behavior. *Archives of Sexual Behavior,**51*(1), 3–37. 10.1007/s10508-020-01745-4 33025291 10.1007/s10508-020-01745-4

[CR15] Dixson, B. J., Dixson, A. F., Bishop, P. J., & Parish, A. (2010a). Human physique and sexual attractiveness in men and women: A New Zealand–U.S. comparative study. *Archives of Sexual Behavior,**39*(3), 798–806. 10.1007/s10508-008-9441-y 19139985 10.1007/s10508-008-9441-y

[CR16] Dixson, B. J., Dixson, A. F., Li, B. G., & Anderson, M. J. (2007a). Studies of human physique and sexual attractiveness: Sexual preferences of men and women in China. *American Journal of Human Biology,**19*(1), 88–95. 17160976 10.1002/ajhb.20584

[CR17] Dixson, B. J., Dixson, A. F., Morgan, B., & Anderson, M. J. (2007b). Human physique and sexual attractiveness: Sexual preferences of men and women in Bakossiland Cameroon. *Archives of Sexual Behavior,**36*(3), 369–375. 17136587 10.1007/s10508-006-9093-8

[CR18] Dixson, B. J., Grimshaw, G. M., Linklater, W. L., & Dixson, A. F. (2010b). Watching the hourglass eye tracking reveals men’s appreciation of the female form. *Human Nature-An Interdisciplinary Biosocial Perspective,**21*(4), 355–370.

[CR19] Dixson, B. J., Grimshaw, G. M., Linklater, W. L., & Dixson, A. F. (2011). Eye-tracking of men’s preferences for waist-to-hip ratio and breast size of women. *Archives of Sexual Behavior,**40*(1), 43–50. 10.1007/s10508-009-9523-5 19688590 10.1007/s10508-009-9523-5

[CR20] Dixson, B. J., Sagata, K., Linklater, W. L., & Dixson, A. F. (2010c). Male preferences for female waist-to-hip ratio and body mass index in the Highlands of Papua New Guinea. *American Journal of Physical Anthropology,**141*(4), 620–625. 19927356 10.1002/ajpa.21181

[CR21] Donohoe, M. L., von Hippel, W., & Brooks, R. C. (2009). Beyond waist-hip ratio: Experimental multivariate evidence that average women’s torsos are most attractive. *Behavioral Ecology,**20*(4), 716–721.

[CR22] Dunson, D. B., Colombo, B., & Baird, D. D. (2002). Changes with age in the level and duration of fertility in the menstrual cycle. *Human Reproduction,**17*(5), 1399–1403. 10.1093/humrep/17.5.1399 11980771 10.1093/humrep/17.5.1399

[CR23] Etcoff, N. L., Stock, S., Haley, L. E., Vickery, S. A., & House, D. M. (2011). Cosmetics as a feature of the extended human phenotype: Modulation of the perception of biologically important facial signals. *PLoS ONE*. 10.1371/journal.pone.0025656 21991328 10.1371/journal.pone.0025656 PMC3185017

[CR24] Fretts, R. C., Schmittdiel, J., McLean, F. H., Usher, R. H., & Goldman, M. B. (1995). Increased maternal age and the risk of fetal death. *New England Journal of Medicine,**333*(15), 953–957. 10.1056/NEJM199510123331501 7666913 10.1056/NEJM199510123331501

[CR25] Furnham, A., Petrides, K. V., & Constantinides, A. (2005). The effects of body mass index and waist-to-hip ratio on ratings of female attractiveness, fecundity, and health. *Personality and Individual Differences,**38*(8), 1823–1834. 10.1016/j.paid.2004.11.011

[CR26] Furnham, A., & Reeves, E. (2006). The relative influence of facial neoteny and waist-to-hip ratio on judgements of female attractiveness and fecundity. *Psychology, Health & Medicine,**11*(2), 129–141. 10.1080/13548500500155982 10.1080/13548500500155982 17129903

[CR27] Furnham, A., Swami, V., & Shah, K. (2006). Body weight, waist-to-hip ratio and breast size correlates of ratings of attractiveness and health. *Personality and Individual Differences,**41*(3), 443–454.

[CR28] Garza, R., Heredia, R. R., & Cieslicka, A. B. (2016). Male and female perception of physical attractiveness: An eye movement study. *Evolutionary Psychology,**14*. 10.1177/1474704916631614

[CR29] Gervais, S. J., Holland, A. M., & Dodd, M. D. (2013). My eyes are up here: The nature of the objectifying gaze toward women. *Sex Roles,**69*(11–12), 557–570.

[CR30] Gervais, S. J., Vescio, T. K., Förster, J., Maass, A., & Suitner, C. (2012). Seeing women as objects: The sexual body part recognition bias. *European Journal of Social Psychology,**42*(6), 743–753. 10.1002/ejsp.1890

[CR31] Henss, R. (2000). Waist-to-hip ratio and female attractiveness. Evidence from photographic stimuli and methodological considerations. *Personality and Individual Differences,**28*(3), 501–513.

[CR32] Hietanen, J. K., & Nummenmaa, L. (2011). The naked truth: The face and body sensitive N170 response is enhanced for nude bodies. *PLoS ONE,**6*(11), Article e24408. 10.1371/journal.pone.0024408 22110574 10.1371/journal.pone.0024408 PMC3217929

[CR33] Hübner, R., & Ufken, E. S. (2024). Curviness is a better predictor of a woman’s body attractiveness than the waist-to-hip ratio. *Scientific Reports,**14*(1), Article 23081. 10.1038/s41598-024-74265-z 39367176 10.1038/s41598-024-74265-z PMC11452730

[CR34] Kiely, J. L., Paneth, N., & Susser, M. (1986). An assessment of the effects of maternal age and parity in different components of perinatal mortality. *American Journal of Epidemiology,**123*(3), 444–454. 10.1093/oxfordjournals.aje.a114259 3946390 10.1093/oxfordjournals.aje.a114259

[CR35] Kościński, K. (2014). Assessment of waist-to-hip ratio attractiveness in women: An anthropometric analysis of digital silhouettes. *Archives of Sexual Behavior,**43*(5), 989–997. 10.1007/s10508-013-0166-1 23975738 10.1007/s10508-013-0166-1 PMC4050298

[CR36] Lassek, W. D., & Gaulin, S. J. C. (2006). Changes in body fat distribution in relation to parity in American women: A covert form of maternal depletion. *American Journal of Physical Anthropology,**131*(2), 295–302. 10.1002/ajpa.20394 16596596 10.1002/ajpa.20394

[CR37] Marcinkowska, U. M., Rantala, M. J., Lee, A. J., Kozlov, M. V., Aavik, T., Cai, H., Contreras-Garduño, J., David, O. A., Kaminski, G., Li,N. P., Onyishi, I. E., Prasai, K., Pazhoohi, F., Prokop, P., Cardozo, S. L. R., Sydney, N.,Taniguchi, H., Krams, I., & Dixson, B. J. W. (2019). Women’s preferences for men’s facial masculinity are strongest under favorable ecological conditions. *Scientific Reports,**9*(1), Article 3387. 10.1038/s41598-019-39350-8 30833635 10.1038/s41598-019-39350-8 PMC6399235

[CR38] Marlowe, F. W., Apicella, C., & Reed, D. (2005). Men’s preferences for women’s profile waist-to-hip ratio in two societies. *Evolution and Human Behavior,**26*(6), 458–468.

[CR39] Menken, J., & Larsen, U. (1986). Fertility rates and aging. In L. M. JR & C. A. Paulsen (Eds.), *Aging, reproduction, and the climacteric* (pp. 147–166). Springer. 10.1007/978-1-4684-5047-7_9

[CR40] Merklinger-Gruchala, A., Jasienska, G., & Kapiszewska, M. (2015). Short interpregnancy interval and low birth weight: A role of parity. *American Journal of Human Biology,**27*(5), 660–666. 10.1002/ajhb.22708 25754897 10.1002/ajhb.22708

[CR41] Mitrovic, A., Tinio, P. P. L., & Leder, H. (2016). Consequences of beauty: effects of rater sex and sexual orientation on the visual exploration and evaluation of attractiveness in real world scenes. *Frontiers in Human Neuroscience,**10*. 10.3389/fnhum.2016.00122 10.3389/fnhum.2016.00122 PMC4800167 27047365

[CR42] Pazhoohi, F., Arantes, J., Kingstone, A., & Pinal, D. (2020). Waist to hip ratio and breast size modulate the processing of female body silhouettes: An EEG study. *Evolution and Human Behavior,**41*(2), 150–169. 10.1016/j.evolhumbehav.2020.01.001

[CR43] Perilloux, H. K., Webster, G. D., & Gaulin, S. J. C. (2010). Signals of genetic quality and maternal investment capacity: The dynamic effects of fluctuating asymmetry and waist-to-hip ratio on men’s ratings of women’s attractiveness. *Social Psychological and Personality Science,**1*(1), 34–42. 10.1177/1948550609349514

[CR44] Platek, S. M., & Singh, D. (2010). Optimal waist-to-hip ratios in women activate neural reward centers in men. *PLoS ONE,**5*. 10.1371/journal.pone.0009042 10.1371/journal.pone.0009042 PMC2816713 20140088

[CR45] Puhl, R. M., & Boland, F. J. (2001). Predicting female physical attractiveness: Waist-to-hip ratio versus thinness. *Psychology, Evolution & Gender,**3*(1), 27–46. 10.1080/14616660110049573

[CR46] Ridley, B. J., Hamamoto, Y., Cornelissen, P. L., Kramer, R. S. S., McCarty, K., & Tovée, M. J. (2025). Perceptual body image tasks require ethnically appropriate stimuli. *Body Image,**53*, Article 101899. 10.1016/j.bodyim.2025.101899 40344951 10.1016/j.bodyim.2025.101899

[CR47] Rilling, J. K., Kaufman, T. L., Smith, E. O., Patel, R., & Worthman, C. M. (2009). Abdominal depth and waist circumference as influential determinants of human female attractiveness. *Evolution and Human Behavior,**30*(1), 21–31.

[CR48] Rozmus-Wrzesinska, M., & Pawlowski, B. (2005). Men’s ratings of female attractiveness are influenced more by changes in female waist size compared with changes in hip size. *Biological Psychology,**68*(3), 299–308. 15620796 10.1016/j.biopsycho.2004.04.007

[CR49] Rupp, H., Librach, G. R., Feipel, N. C., Ketterson, E. D., Sengelaub, D. R., & Heiman, J. R. (2009). Partner status influences women’s interest in the opposite sex. *Human Nature,**20*(1), 93–104. 10.1007/s12110-009-9056-6 20161078 10.1007/s12110-009-9056-6 PMC2743495

[CR50] Schützwohl, A. (2006). Judging female figures: A new methodological approach to male attractiveness judgments of female waist-to-hip ratio. *Biological Psychology,**71*(2), 223–229. 10.1016/j.biopsycho.2005.04.005 16019126 10.1016/j.biopsycho.2005.04.005

[CR51] Seidell, J., Baanders-van, E. H., & Ouwehand, I. (1990). Fat distribution in relation to age, degree of obesity, smoking habits, parity and estrogen use: A cross-sectional study in 11,825 Dutch women participating in the DOM-project. *International Journal of Obesity,**14*(9), 753–761. 2228408

[CR52] Shiramizu, V., Docherty, C., DeBruine, L. M., & Jones, B. C. (2020). Sexual orientation predicts men’s preferences for sexually dimorphic face-shape characteristics: A replication study. *PLoS ONE,**15*(11), Article e0242262. 10.1371/journal.pone.0242262 33186368 10.1371/journal.pone.0242262 PMC7665808

[CR53] Sidari, M. J., Lee, A. J., Murphy, S. C., Sherlock, J. M., Dixson, B. J. W., & Zietsch, B. P. (2021). Preferences for sexually dimorphic body characteristics revealed in a large sample of speed daters. *Social Psychological and Personality Science,**12*, 225–236 . 10.1177/1948550619882925

[CR54] Singh, D. (1993a). Body shape and women’s attractiveness - The critical role of waist-to-hip ratio. *Human Nature-an Interdisciplinary Biosocial Perspective,**4*(3), 297–321. 24214368 10.1007/BF02692203

[CR55] Singh, D. (1993b). Adaptive significance of female physical attractiveness - Role of waist-to-hip ratio. *Journal of Personality and Social Psychology,**65*(2), 293–307. 8366421 10.1037//0022-3514.65.2.293

[CR56] Singh, D. (1994). Is thin really beautiful and good a Relationship between waist-to-hip ratio (WHR) and female attractiveness. *Personality and Individual Differences,**16*(1), 123–132.

[CR57] Singh, D., & Randall, P. K. (2007). Beauty is in the eye of the plastic surgeon: Waist-hip ratio (WHR) and women’s attractiveness. *Personality and Individual Differences,**43*(2), 329–340. 10.1016/j.paid.2006.12.003

[CR58] Sorokowski, P., Kościński, K., Sorokowska, A., & Huanca, T. (2014). Preference for women’s body mass and waist-to-hip ratio in Tsimane’men of the Bolivian Amazon: Biological and cultural determinants. *PLoS ONE,**9*(8), Article e105468. 25148034 10.1371/journal.pone.0105468 PMC4141791

[CR59] Steele, V. (2001). *The corset: A cultural history*. Yale University Press.

[CR60] Sugiyama, L. S. (2004). Is beauty in the context-sensitive adaptations of the beholder? Shiwiar use of waist-to-hip ratio in assessments of female mate value. *Evolution and Human Behavior,**25*(1), 51–62.

[CR61] Suschinsky, K. D., Elias, L. J., & Krupp, D. B. (2007). Looking for Ms. Right: Allocating attention to facilitate mate choice decisions. *Evolutionary Psychology,**5*(2), 428–441.

[CR62] Swami, V., Caprario, C., Tovee, M. J., & Furnham, A. (2006). Female physical attractiveness in Britain and Japan: A cross-cultural study. *European Journal of Personality,**20*(1), 69–81.

[CR63] Swami, V., Grant, N., Furnham, A., & McManus, I. C. (2007a). Perfectly formed? The effect of manipulating the waist-to-hip ratios of famous paintings and sculptures. *Imagination, Cognition and Personality,**27*(1), 47–62.

[CR64] Swami, V., Gray, M., & Furnham, A. (2007b). The female nude in Rubens: Disconfirmatory evidence of the waist-to-hip ratio hypothesis of female physical attractivenes. *Imagination, Cognition and Personality,**26*(1), 139–147.

[CR65] Swami, V., Jones, J., Einon, D., & Furnham, A. (2009). Men’s preferences for women’s profile waist-to-hip ratio, breast size, and ethnic group in Britain and South Africa. *British Journal of Psychology,**100*, 313–325. 18625082 10.1348/000712608X329525

[CR66] Swami, V., & Tovée, M. J. (2005). Female physical attractiveness in Britain and Malaysia: A cross-cultural study. *Body Image,**2*(2), 115–128. 10.1016/j.bodyim.2005.02.002 18089180 10.1016/j.bodyim.2005.02.002

[CR67] Swami, V., & Tovée, M. J. (2006). Does hunger influence judgments of female physical attractiveness? *British Journal of Psychology,**97*(3), 353–363. 10.1348/000712605X80713 16848948 10.1348/000712605X80713

[CR68] Tassinary, L. G., & Hansen, K. A. (1998). A critical test of the waist-to-hip-ratio hypothesis of female physical attractiveness. *Psychological Science,**9*(2), 150–155.

[CR69] Tovée, M. J., & Cornelissen, P. L. (2001). Female and male perceptions of female physical attractiveness in front-view and profile. *British Journal of Psychology,**92*(Pt 2), 391–402. 11802880

[CR70] Tovée, M. J., Hancock, P. J. B., Mahmoodi, S., Singleton, B. R. R., & Cornelissen, P. L. (2002). Human female attractiveness: Waveform analysis of Body shape. *Proceedings of the Royal Society B: Biological Sciences,**269*(1506), 2205–2213. 10.1098/rspb.2002.2133 10.1098/rspb.2002.2133 PMC1691155 12427313

[CR71] Tovée, M. J., Maisey, D. S., Emery, J. L., & Cornelissen, P. L. (1999). Visual cues to female physical attractiveness. *Proceedings of the Royal Society of London. Series B: Biological Sciences,**266*(1415), 211–218. 10.1098/rspb.1999.0624 PMC1689653 10097394

[CR72] Veblen, T. (1965). *The theory of the leisure class*. Kelley.

[CR73] Vigarello, G. (2004). *Histoire de la beauté: Le corps et l’art d’embellir de la Renaissance à nos jours*. SEUIL.

[CR74] Waugh, N. (2015). *Corsets and crinolines*. Routledge. 10.4324/9781315060071

[CR75] Weeden, J., & Sabini, J. (2005). Physical attractiveness and health in Western societies: A review. *Psychological Bulletin,**131*(5), 635–653. 10.1037/0033-2909.131.5.635 16187849 10.1037/0033-2909.131.5.635

[CR76] Wells, J. C. K., Charoensiriwath, S., & Treleaven, P. (2011). Reproduction, aging, and body shape by three-dimensional photonic scanning in Thai men and women. *American Journal of Human Biology,**23*(3), 291–298. 10.1002/ajhb.21151 21387458 10.1002/ajhb.21151

[CR77] Wesselink, A. K., Rothman, K. J., Hatch, E. E., Mikkelsen, E. M., Sørensen, H. T., & Wise, L. A. (2017). Age and fecundability in a North American preconception cohort study. *American Journal of Obstetrics and Gynecology,**217*(6), 667.e1-667.e8. 10.1016/j.ajog.2017.09.002 28917614 10.1016/j.ajog.2017.09.002 PMC5712257

[CR78] Wetsman, A., & Marlowe, F. W. (1999). How universal are preferences for female waist-to-hip ratios? Evidence from the Hadza of Tanzania. *Evolution and Human Behavior,**20*(4), 219–228.

[CR79] White, H., Chroust, A., Jubran, R., Heck, A., & Bhatt, R. S. (2020). Waist-to-hip ratio sensitivity in early infancy. *Infant and Child Development,**29*(3), Article e2170. 10.1002/icd.2170 34385889 10.1002/icd.2170 PMC8356647

[CR80] Wilson, J. M. B., Tripp, D. A., & Boland, F. J. (2005). The relative contributions of waist-to-hip ratio and body mass index to judgements of attractiveness. *Sexualities, Evolution & Gender,**7*, 245–267. 10.1080/14616660500238769

[CR81] Yu, D. W., & Shepard, G. H., Jr. (1998). Is beauty in the eye of the beholder? *Nature,**396*, 321–322. 9845067 10.1038/24512

[CR82] Zotto, M. D., & Pegna, A. J. (2017). Electrophysiological evidence of perceived sexual attractiveness for human female bodies varying in waist-to-hip ratio. *Cognitive, Affective, & Behavioral Neuroscience,**17*(3), 577–591. 10.3758/s13415-017-0498-8 10.3758/s13415-017-0498-8 28315140

